# Marked mitochondrial genetic variation in individuals and populations of the carcinogenic liver fluke *Clonorchis sinensis*

**DOI:** 10.1371/journal.pntd.0008480

**Published:** 2020-08-19

**Authors:** Liina Kinkar, Pasi K. Korhonen, Daxi Wang, Xing-Quan Zhu, Galina N. Chelomina, Tao Wang, Ross S. Hall, Anson V. Koehler, Ivon Harliwong, Bicheng Yang, J. Lynn Fink, Neil D. Young, Robin B. Gasser

**Affiliations:** 1 Department of Veterinary Biosciences, Melbourne Veterinary School, Faculty of Veterinary and Agricultural Sciences, The University of Melbourne, Parkville, Victoria, Australia; 2 BGI International, Shenzhen, China; 3 State Key Laboratory of Veterinary Etiological Biology, Key Laboratory of Veterinary Parasitology of Gansu Province, Lanzhou Veterinary Research Institute, Chinese Academy of Agricultural Sciences, Lanzhou, Gansu Province, China; 4 Department of Parasitology, Federal Scientific Center of the East Asia Terrestrial Biodiversity FEB RAS, Vladivostok, Russia; 5 BGI Australia, Herston, Queensland, Australia; James Cook University, AUSTRALIA

## Abstract

Clonorchiasis is a neglected tropical disease caused by the Chinese liver fluke, *Clonorchis sinensis*, and is often associated with a malignant form of bile duct cancer (cholangiocarcinoma). Although some aspects of the epidemiology of clonorchiasis are understood, little is known about the genetics of *C*. *sinensis* populations. Here, we conducted a comprehensive genetic exploration of *C*. *sinensis* from endemic geographic regions using complete mitochondrial protein gene sets. Genomic DNA samples from *C*. *sinensis* individuals (n = 183) collected from cats and dogs in China (provinces of Guangdong, Guangxi, Hunan, Heilongjiang and Jilin) as well as from rats infected with metacercariae from cyprinid fish from the Russian Far East (Primorsky Krai region) were deep sequenced using the BGISEQ-500 platform. Informatic analyses of mitochondrial protein gene data sets revealed marked genetic variation within *C*. *sinensis*; significant variation was identified within and among individual worms from distinct geographical locations. No clear affiliation with a particular location or host species was evident, suggesting a high rate of dispersal of the parasite across endemic regions. The present work provides a foundation for future biological, epidemiological and ecological studies using mitochondrial protein gene data sets, which could aid in elucidating associations between particular *C*. *sinensis* genotypes/haplotypes and the pathogenesis or severity of clonorchiasis and its complications (including cholangiocarcinoma) in humans.

## Author summary

Clonorchiasis is an important neglected tropical disease caused by the Chinese liver fluke, *Clonorchis sinensis*, which can induce malignant bile duct cancer (cholangiocarcinoma). Little precise information is available on the biology, epidemiology and population genetics of *C*. *sinensis*. For this reason, we explored here the genetic composition of *C*. *sinensis* populations in distinct endemic areas in China and Russia. Using a deep sequencing-informatic approach, we revealed marked mitochondrial genetic variation within and between individuals and populations of *C*. *sinensis*, with no particular affiliation with geographic or host origin. These molecular findings and the methodology established should underpin future genetic studies of *C*. *sinensis* causing human clonorchiasis and associated complications (cancer) as well as transmission patterns in endemic regions.

## Introduction

Eleven pathogens are recognised to cause one fifth of all human cancers worldwide [1]. One of them is the Chinese liver fluke, *Clonorchis sinensis* (phylum Platyhelminthes; class Trematoda; family Opisthorchiidae). Chronic infection with this fluke can induce cholangiocarcinoma in humans–a malignant and fatal form of bile duct cancer [2].

The disease (clonorchiasis) caused by *C*. *sinensis* is of major public health concern in parts of Asia [3–5]; ~ 15 million people are infected, and the highest endemicity is in South Korea, Vietnam, China and the Russian Far East [6, 7]. Most of these people (~ 13 million) live within provinces in the southern (Guangdong, Guangxi, and Hunan) and north-eastern (Heilongjiang and Jilin) parts of China [4, 6–8]. In endemic areas, millions of tourists per annum are also at risk of infection, as they often consume local delicacies made from raw fish or shrimp [9]. Humans and other piscivorous mammals (e.g., canids and felids) are definitive hosts, and acquire infection by eating raw freshwater (cyprinid) fish or shrimp containing encysted larvae (= metacercariae) which ultimately establish as adult worms in the biliary system [7, 8]. Here, eggs produced by adult *C*. *sinensis* are released into aquatic environments to infect small snail intermediate hosts (order Mesogastropoda); larval stages (cercariae) released from the snails then usually infect fish (family Cyprinidae) and encyst as metacercariae.

An epidemiological factor contributing significantly to human clonorchiasis relates to human behaviour. In some endemic regions, such as in the Chinese provinces of Guangdong and Guangxi, it is commonplace in fish-producing areas for toilets to be built adjacent to fish ponds, so that human faeces containing *C*. *sinensis* eggs enter fish ponds; such eggs are thus accessible to snail hosts, and then infect cyprinid fish [8]. In such regions, the often high dependence on aquaculture for food; fish producers’ unawareness of the life cycle of *C*. *sinensis*; the intimate involvement of canids and felids in the transmission; and environmental and climatic (temperature and humidity) conditions all contribute significantly to the endemicity of clonorchiasis [6–8, 10–12].

Although these aspects of the epidemiology of clonorchiasis (transmission and prevalence) are relatively well understood [4, 8], there is little comprehensive information on the genetics of *C*. *sinensis* and what impact genetic variation within and among populations has on the biology and transmission of *C*. *sinensis* infection and the severity of clonorchiasis and/or associated cancer. Published population genetic studies conducted using selected genetic markers, including the internal transcribed spacers (ITS-1 and/or ITS-2) of nuclear ribosomal DNA and the mitochondrial *cox*1 gene regions, have indicated variable levels of genetic variation and sub-structuring within *C*. *sinensis* populations [13–22]. Other studies have reported draft nuclear genomes of *C*. *sinensis* from Korea and China [23, 24] and four mitochondrial genomes of individual isolates from Korea, China and the Russian Far East [25–27].

Given the known utility of mitochondrial genomic data sets for investigating genetic variation within flatworm taxa [28, 29], encoded protein genes provide a sound basis for comprehensive population genetic studies. For this reason, we undertook the first detailed population genetic exploration of *C*. *sinensis* from high-endemicity regions in China and the Russian Far East using complete mitochondrial protein-encoding gene data sets. This work provides a foundation for future biological, epidemiological and ecological studies of this parasite.

## Methods

### Ethics statement

The collection of *C*. *sinensis* in China was approved by the South China Agricultural University [30], following the Animal Ethics Guidelines of the People’s Republic of China. The production of *C*. *sinensis* in rats was approved by the Ethics Committee for Animal Experimentation of the Institute of Biology and Soil Science, Russia (permit no. 3_02.06.2011; [21]).

### Parasite material

Adult *C*. *sinensis* worms (n = 183) were collected from China and the Russian Far East–two regions endemic for *C*. *sinensis* [4] (Table 1 and S1 Table). Specimens from China were from the livers of naturally infected cats or dogs originating from the provinces of Guangdong, Guangxi, Hunan, Heilongjiang and Jilin. Biliary ducts were flushed with physiological saline, intact *C*. *sinensis* worms collected, washed extensively in physiological saline and then fixed in 70% (v/v) ethanol. Specimens from the Russian Far East (Primorsky Krai region) were from rats experimentally infected with metacercariae collected from naturally infected freshwater fish (family Cyprinidae). All samples were fixed in ethanol and stored at -80°C until further use.

**Table 1:** Geographical origin and host species of the 183 specimens of *Clonorchis sinensis* studied herein.

Origin^a^	Dog	Cat	Cyprinid fish^b^	Totals
**China**				
Guangdong	70	61		131
Guangxi		13		13
Hunan	5			5
Heilongjiang	9			9
Jilin	13			13
**Russia**				
Primorsky Krai			12	12
**Totals:**	97	74	12	183

^a^ Heilongjiang and Jilin are northern provinces, and Guangdong, Guangxi and Hunan are southern provinces in China.

^b^ Specimens from rats experimentally infected with metacercariae from cyprinid fish.

### DNA isolation, amplification and sequencing of mitochondrial protein genes

The most posterior tip (2–4 mm) of each of the 183 adult specimens of *C*. *sinensis* was excised under a dissecting microscope (10× magnification) using a sterile scalpel, in order to exclude eggs from the reproductive tract. Genomic DNA was extracted from each of these tips using the DNeasy Blood & Tissue Kit (Qiagen), following the manufacturer’s protocol. DNA amounts were determined using a Qubit fluorometer dsDNA HS kit (Invitrogen). Subsequently, individual DNA samples (representing individual *C*. *sinensis* adults) were whole genome-amplified using the REPLI-g Mini Kit (QIAGEN; cat. no. 150025), and individual genomic DNA libraries constructed using the DNA Library Prep Kit (MGI, item no. 1000002505), employing a well-established protocol [31], and then sequenced (100 bp paired-end reads) using the BGISEQ-500 platform (BGI Australia).

### Detection of unambiguous (i.e. fixed) nucleotide variations

For each of the 183 *C*. *sinensis* specimens, raw DNA sequence data in the FASTQ format [32] were filtered for quality (Phred quality score cut-off: 20) and trimmed using the program trimmomatic v.0.38 [33]. Filtered read-pairs were mapped to a reference mitochondrial genome of *C*. *sinensis* (GenBank accession no. KY564177–a sequence without polymorphism; [27]) using the Burrows-Wheeler Aligner (BWA) v.0.7.8 [34]. For each sample, single nucleotide variations (SNVs) at individual positions and insertion/deletion events (indels) in relation to this reference were recorded using a workflow in the Genome Analysis Toolkit (GATK v.4.0.8.1; [35]), and merged into one ‘variant call format’ (VCF) file that listed all variable sites for all samples. This file was used to produce a consensus FASTA file containing the sequence data for each individual *C*. *sinensis* specimen using BCFtools v.1.9 [36]. The open reading frame (ORF) of each protein gene was verified and conceptually translated using the program Geneious v.11.1.5 [37] employing the mitochondrial genetic code for echinoderms and flatworms (cf. [29]; https://www.ncbi.nlm.nih.gov/Taxonomy/Utils/wprintgc.cgi; translation_table 9). For each of the 183 *C*. *sinensis* specimens, the nucleotide sequences of all 12 protein genes and conceptually translated amino acid sequences were defined and deposited in the GenBank database (accession nos. MT292110–MT292292).

### Detection of nucleotide polymorphism within individual *C*. *sinensis* specimens

Duplicates were removed from the BWA-mapped sequence data using Picard v.2.0.1 (http://broadinstitute.github.io/picard). Subsequently, SAMtools v.1.9 [38] was used to compile an mpileup file; this file provided a summary of nucleotide coverage at each position following mapping to the reference sequence. For each specimen, any multiple-nucleotide variation (i.e. sequence polymorphism or microheterogeneity) at individual positions with respect to the reference was identified using the program VarScan v.2.4.3 [39]. The percentages of individual bases contributing to each polymorphic position (i.e. 0 to 100%) were recorded in a VCF file; the minimum base-contribution for a position to be recorded as polymorphic was ≥ 10%, with a minimum of 1400 nucleotides mapping to such a position. Each polymorphic position recorded was verified by eye using the integrative genomics viewer (IGV) v.2.5.2 [40].

### Population genetic analyses

All 12 protein gene sequences of all 183 *C*. *sinensis* individuals produced here (excluding polymorphic positions) and of four respective, published sequences (accession nos. KY564177 from Korea; JF729304 from Korea, JF729303 from China; FJ381664 from Russia; [25–27]) were aligned, as were their amino acid sequences.

The aligned nucleotide sequence data (Mendeley Data doi: 10.17632/dj4ybp77jw.2) were subjected to phylogenetic analyses, employing *Opisthorchis felineus* (family Opisthorchiidae) (accession no. EU921260; [25]) as an outgroup–as required. First, a Bayesian (BI) tree was built using Monte Carlo Markov Chain (MCMC) analysis [41, 42] in MrBayes v.3.2.6 [43]. The general time-reversible model, employing a gamma-distribution and a proportion of invariable sites (GTR+I+G; [44, 45]), was established to provide the best-fit model of sequence evolution using the program PartitionFinder v.2.1.1 [46]. Posterior probability (pp) values were calculated by running 40 million generations, with 25% discarded as non-converged burn-in. Second, a maximum likelihood (ML) method was used to infer nodal support values for the BI tree using the program RAxML v.8.2.9 [47] employing 1000 bootstrap replicates. Third, a median-joining (MJ) network [48] was constructed using the program PopART [49], with epsilon set at zero. The aligned amino acid sequences were used to further assess phylogenetic clustering and support.

Subsequently, population genetic diversity and differentiation were assessed in relation to geographical origin or known host species. Population diversity indices (number of haplotypic sequences, haplotype diversity, nucleotide diversity, and mean number of nucleotide variations between haplotypic sequences) were estimated using DnaSP v.6.12.03 [50]. To estimate the degree of genetic differentiation between *C*. *sinensis* subpopulations (in relation to geographic origin or definitive host species with natural *C*. *sinensis* infection), the pairwise fixation index (*F*_ST_) was calculated using Arlequin v.3.5.2.2 [51]. To assess patterns of nucleotide variation in all mitochondrial protein genes, a sliding window analysis of nucleotide diversity (steps of 10 bp over 300 bp-windows) was performed using the PopGenome package [52] in the R software environment [53]. For each data set, nucleotide diversity values were plotted against midpoint positions in each window using the R package ggplot2 [54].

## Results

### Sequence data sets and mapping results

A large amount of high-quality, trimmed short-read mitochondrial genomic data (mean: 665 Mb) for each of the 183 *C*. *sinensis* specimens was mapped to the reference mitochondrial genome sequence (accession no. KY564177), revealing substantial depth (median: 7268 nucleotides) at each position in each of the 12 protein genes. This mapping showed that most specimens (n = 164) had haplotypic sequences (without polymorphism within individuals), while a small subset of *C*. *sinensis* specimens (n = 19) had sequences with ≤ 3 polymorphic positions (Table 2). Such specimens originated mostly from Guangdong (n = 17), whereas single specimens were from Guangxi and Hunan.

**Table 2:** List of polymorphic positions in the sequences of five mitochondrial protein genes derived from 19 of a total of 183 specimens of *Clonorchis sinensis*.

Gene	Position in the reference sequence^a^	Base in the reference sequence	Polymorphism recorded and base-contribution (in %)	Codon position	Specimen nos.
*cox*3	96	T	T (71) and C (29)	3 (synonymous)	21^b^
*cox*3	191	A	A (89) and G (11)	2 (non-synonymous)	58, 59, 60 and 61
*cox*3	219	A	A (87–90) and G (10–13)	3 (synonymous)	58, 60 and 61
*cox*3	229	T	T (86–90) and G (10–14)	1 (non-synonymous)	34, 35, 36, 37, 58, 60, 61 and 64
*cox*3	330	C	C (84) and T (16)	3 (synonymous)	12
*nad*3	6718	A	A (52) and G (48)	1 (non-synonymous)	189^b^
*cox*1	8152	T	T (90) and A (10)	3 (synonymous)	160
*cox*1	8414	T	T (86–88) and G (12–14)	1 (non-synonymous)	75, 100 and 125
*nad*6	11340	T	T (52) and A (48)	2 (non-synonymous)	177^b^
*nad*5	12832	A	A (75) and G (25)	1 (non-synonymous)	30^b^
*nad*5	13009	A	A (82) and G (18)	1 (non-synonymous)	154

^a^ Sequence with GenBank accession no. KY564177 [27].

^b^ Base-contribution of the minor-frequency nucleotide: > 20%.

### Marked mitochondrial genetic variation within *C*. *sinensis*

An analysis of haplotypic diversity indicated marked genetic variation among the 183 *C*. *sinensis* specimens (Hd = 0.998; Table 3), and within all sub-populations relating to geographical provenance (0.939 to 1.000) and host species (cat, dog or cyprinid fish) (0.939 to 1.000). The number of nucleotide differences among haplotypes/individuals (upon pairwise comparison) varied from 1 to 75, with a mean of 36 (Table 3). Nucleotide diversity values indicated that genetic variation within *C*. *sinensis* in the Chinese provinces of Jilin (π = 0.00223; Table 3) and Hunan (0.00167) was lower than in other geographical locations (ranging from 0.00300 to 0.00384). *C*. *sinensis* specimens originating from cats, dogs or cyprinid fish displayed similar levels of genetic diversity, with nucleotide diversity values ranging from 0.00344 to 0.00364. Having established marked variation among haplotypes, we proceeded to investigate the genetic relationships of haplotypes from different geographic provenances and definitive host species (cats and dogs).

**Table 3:** Diversity indices calculated for the 183 *Clonorchis sinensis* specimens used in the present study.

	No. of isolates	No. of haplotypes	Haplotype diversity (Hd) ± standard deviation	Nucleotide diversity (π) ± standard deviation	Average no. of nucleotide differences
Complete data set	183	162	0.998 ± 0.001	0.00356 ± 0.00009	36
**Regions**					
‘North’ (northern China and Russia)	34	29	0.989 ± 0.010	0.00325 ± 0.00027	33
Northern China	22	21	0.996 ± 0.015	0.00300 ± 0.00035	30
Russia	12	9	0.939 ± 0.058	0.00350 ± 0.00045	36
‘South’ (southern China)	149	133	0.998 ± 0.001	0.00359 ± 0.00010	36
**Provinces in China**					
Heilongjiang	9	9	1.000 ± 0.052	0.00384 ± 0.00053	39
Jilin	13	13	1.000 ± 0.030	0.00223 ± 0.00031	23
Guangdong	131	119	0.998 ± 0.001	0.00346 ± 0.00009	35
Guangxi	13	13	1.000 ± 0.030	0.00351 ± 0.00034	36
Hunan	5	5	1.000 ± 0.126	0.00167 ± 0.00029	17
**Host species**					
Cat	74	73	1.000 ± 0.002	0.00364 ± 0.00012	37
Dog	97	88	0.998 ± 0.002	0.00344 ± 0.00014	35
Cyprinid fish^a^	12	9	0.939 ± 0.058	0.00350 ± 0.00045	36

^a^ Specimens from rats experimentally infected with metacercariae from naturally infected cyprinid fish.

### Affiliation of mitochondrial haplogroups with geographical provenance or host species

**Fig 1:**
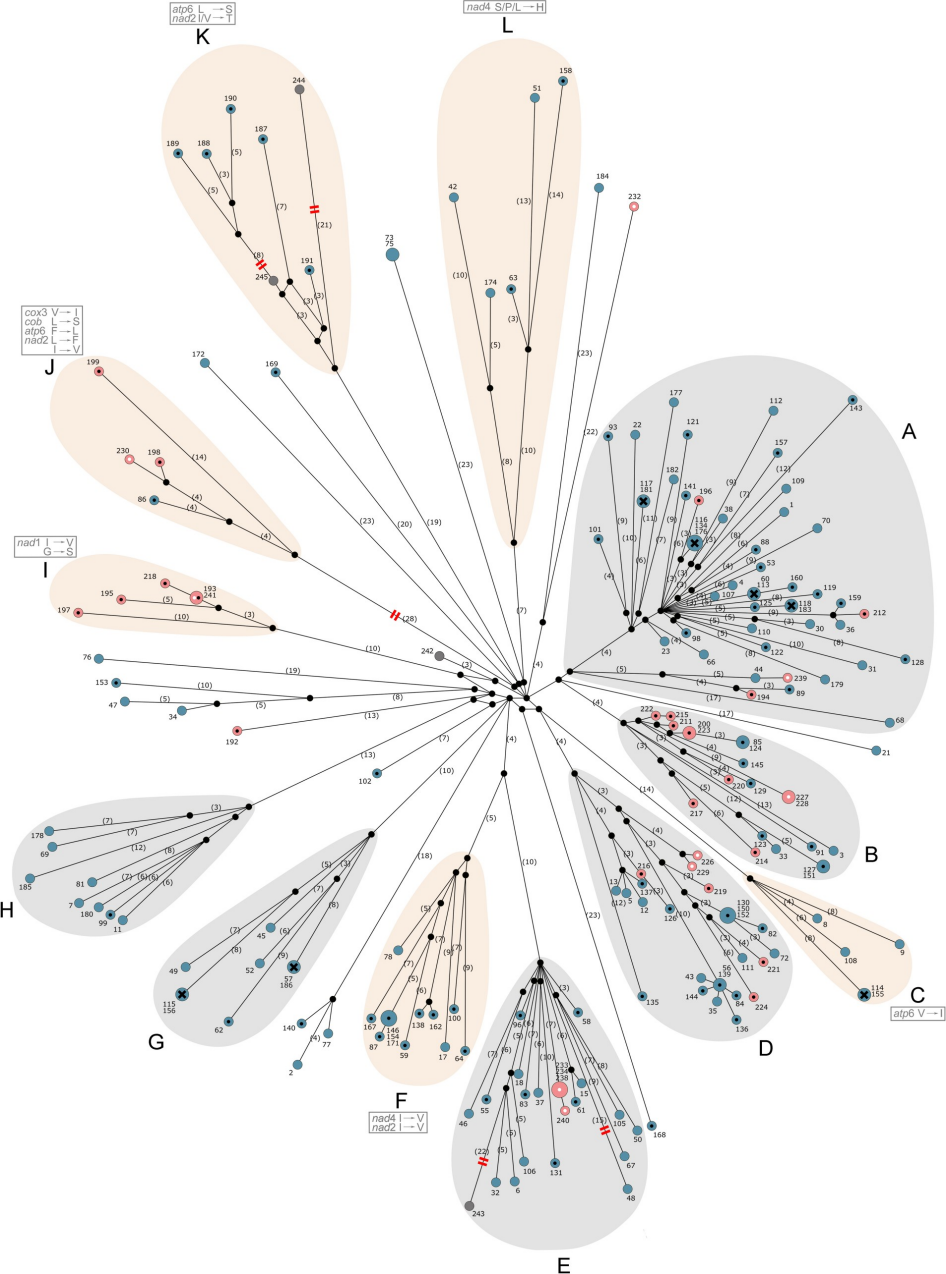
Median-joining haplotype network constructed using concatenated sequences for the 12 mitochondrial protein genes representing 183 *Clonorchis sinensis* specimens and four published mitochondrial genomes (GenBank accession nos. JF729303, FJ381664, JF729304 and KY564177). Pink and blue circles represent haplotypic sequences produced in the present study; blue represents specimens from southern China (Guangdong, Hunan and Guangxi provinces), pink represents northern China (Heilongjiang and Jilin provinces) and the Russian Far East (Primorsky Krai region). Host species are indicated with a black dot (dog), solid colour (cat), black cross (cat and dog) or white dot (rats experimentally infected with metacercariae from cyprinid fish). Dark grey circles are haplotypic sequences from GenBank–haplotype 242 represents a *C*. *sinensis* specimen from a hamster experimentally infected with metacercariae obtained from cyprinid fish from Russia (FJ381664; [25]), haplotype 243 is from a cat from China (accession no. JF729303; [26]), haplotype 244 is from a cat from Korea (JF729304; [26]), and haplotype 245 is from a hamster experimentally infected with metacercariae obtained from cyprinid fish from South Korea (KY564177; [27]). Black circles at nodes represent median vectors (i.e. haplotypes not sampled, or extinct). Numbers of mutations (≥ 3) between haplotypes and/or median vectors in parentheses. Specimen numbers–not in parantheses (see S1 Table). Black lines indicate network edges, the lengths of which are proportional to the number of mutations (note that several mutations are shared by some edges in the network). Red lines on branches indicate reduced edge-lengths. Clusters A to L indicate individual haplogroups; a grey outline indicates a haplogroup supported by nucleotide sequence data; a beige outline indicates a haplogroup supported by both nucleotide and amino acid sequence data. Unique amino acid substitutions in protein sequences characteristic of all haplotypes of a respective cluster are indicated by grey boxes; amino acid abbreviations: V–valine, I–isoleucine, G–glycine, S–serine, L–leucine, F–phenylalanine, P–proline, H–histidine, T–threonine.

Most haplotypes defined herein clustered into 12 genetically closely-related groups (haplogroups A to L), with more than 3 haplotypes per haplogroup (Fig 1). Haplogroups were well-supported by BI and ML analyses, with near-absolute nodal support values (pp > 0.95 and bs ≥ 0.93), with the exception of haplogroup A, for which bs was 0.6 (S1 Fig). *F*_ST_ values of 0.43692 to 0.80709 (S2 Table) indicated a clear genetic differentiation of individual haplogroups from one other. Analyses of amino acid data sets further supported the clustering of six haplogroups (C, F, I, J, K and L; Fig 1).

**Fig 2:**
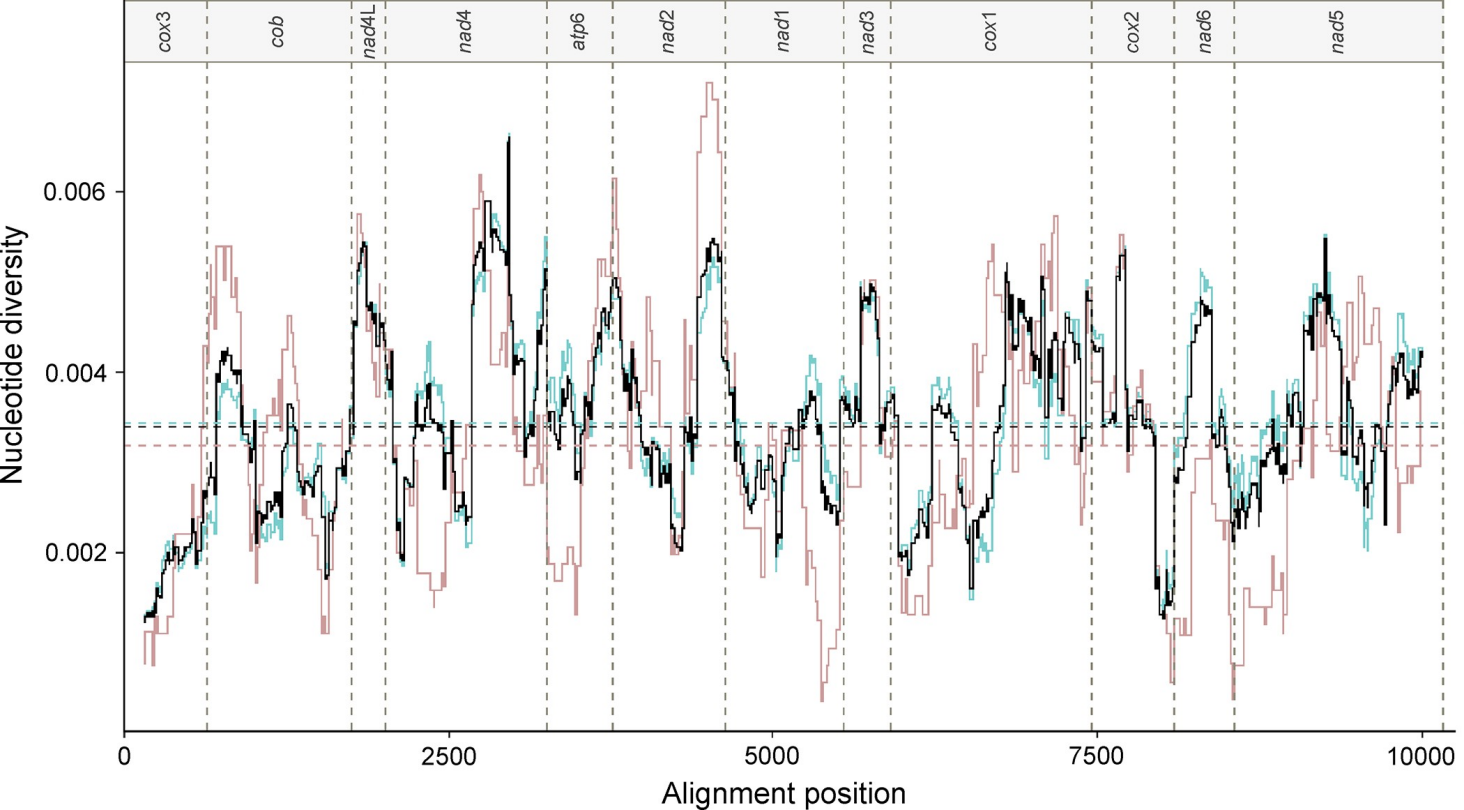
Results from sliding window analysis of nucleotide diversity. Nucleotide diversity values calculated based on all 12 concatenated mitochondrial protein gene sequences for: (i) all 183 *Clonorchis sinensis* specimens studied (black line); (ii) 149 specimens from the ‘south’ (Chinese provinces Guangdong, Guangxi and Hunan; blue line); and (iii) 34 specimens from the ‘north’ (Primorsky Krai region in the Russian Far East, and Chinese provinces Heilongjiang and Jilin; pink line).

Subsequently, we proceeded to investigate whether there was an affiliation between haplogroups and geographical provenance. First, we tested the hypothesis that there is a genetic distinction between the ‘south’ and the ‘north’; however, no unequivocal genetic differentiation could be identified. Although haplogroup I comprised haplotypes exclusive to the ‘north’ (Primorsky Krai, Heilongijang and Jilin) and haplogroups C, F, G, H and L comprised haplotypes that were only in the ‘south’ (Guangdong and Guangxi), for several clusters (A, B, D, E and J) ‘southern’ and ‘northern’ haplotypes grouped together, indicating their close genetic relationship. This lack of differentiation was well-supported by low *F*_ST_ values between ‘north’ and ‘south’ (0.02893, *p* < 0.00001). The sliding window analysis of nucleotide diversity also indicated a lack of genetic distinctiveness in *C*. *sinensis* between the two geographic regions. We detected similar patterns of genetic diversity between the two subpopulations, with minor differences in the range of values for particular genes, being often more pronounced for specimens originating from the ‘north’ (Fig 2). Subsequently, we subdivided northern specimens into northern China and the Russian Far East, to assess whether these subpopulations might be genetically differentiated. However, *F*_ST_ values did not indicate a genetic differentiation in *C*. *sinensis* between these regions–low values were recorded between southern China and Russia (0.04095, *p* < 0.01), southern China and northern China (0.03542, *p* < 0.001), and the Russian Far East and northern China (0.04969, *p* < 0.05). Nonetheless, we did detect some evidence of geographical sub-structuring. Within haplogroup K, two samples from Korea represented by sequences with accession nos. KY564177 and JF729304 clustered together, as did all five specimens from Hunan. *F*_ST_ values (0.42205 to 0.56811; S3 Table) revealed a clear genetic distinction between subpopulations from Hunan, other Chinese provinces and the Russian Far East (Primorsky Krai region), with significantly lower *F*_ST_ values (0.02364 to 0.0999; S3 Table) between other provinces in China and Primorsky Krai in Russia–upon pairwise comparison.

Separate analyses did not find evidence for an affiliation between particular haplogroups and known definitive host species, as all genetic clusters, except haplogroups I and J, represented specimens from cats and dogs, and some specimens from cats and dog hosts were genetically identical (i.e. haplogroups A, C and G; see Fig 1). This finding was further supported by low values of *F*_ST_ between the two host species (0.01023, *p* < 0.01).

## Discussion

Here, we discovered marked mitochondrial genetic variation within *C*. *sinensis*. This variation in protein genes was present within individual worms, between individual worms, and within populations from distinct geographical locations and different species of hosts. These results should have implications for future explorations of the biology, epidemiology and ecology of this parasite.

Nucleotide polymorphism (microheterogeneity) within some worms likely represents heteroplasmy as a result of multiple, distinct mitochondrial genomes being present within an individual. This interpretation is supported by the high sequencing depth achieved (the median value being 7268). However, it is possible that other factors, such as nuclear DNA of mitochondrial origin (called nuclear mitochondrial sequences, NUMTs; [55]) or sequencing error might cause ‘background’ issues. NUMTs can occur as a result of the transposition of mitochondrial sequence tracts to the nuclear genome; although their prevalence has not been investigated in *C*. *sinensis*, such DNA elements have been identified or characterised in a range of organisms, such as fungi, plants and metazoans including flatworms [55, 56]. In the sequencing procedure employed herein, such sequences or mutants thereof might have been co-amplified from homologous mitochondrial sequences, being reflected as polymorphism at a low frequency [57], given that mitochondrial sequences usually have a higher copy number than NUMTs in the nuclear genome [58]. Sequencing errors may also masquerade as polymorphism, and would occur randomly at low level frequency [59, 60]. To distinguish authentic polymorphism (i.e. heteroplasmy) from such ‘background’, a detection threshold is commonly applied (cf. [61]). Thresholds in the range of 10% to 20% have been shown to minimise the risk of ‘false attribution’ in the event that NUMTs or sequencing errors are present [61, 62]. Using detection thresholds of 10% and 20%, genuine polymorphism (heteroplasmy) was inferred here in 19 and 4 individual *C*. *sinensis* specimens, respectively (Table 2).

Heteroplasmy is relatively common in mitochondrial genomes of eukaryotes (e.g., [63–65]); however, the biological, evolutionary and molecular mechanisms leading to it have not been explored in-depth in helminths. On one hand, heteroplasmy is thought to occur as an intermediate state of a mutation before becoming fixed in the mitochondrial genome. On the other hand, multiple populations of mitochondrial haplotypes within an individual might be positively selected for, given that complex tissue-specific differences in mitochondria and their genomes are known to occur [66, 67]. Such genetic variation within individuals could be acquired *de novo*, inherited maternally or, in rare cases, inherited paternally [68–70]. Although mitochondrial genomes are believed to be maternally inherited, paternal leakage of mitochondria during fertilisation has been reported in metazoans [68, 69, 71, 72] and may occur in *C*. *sinensis*. Assuming that out-crossing occurs in *C*. *sinensis*, or that mitochondrial genomes in spermatozoa and oocytes of hermaphroditic individuals display some level of genetic variation, a small number of mitochondria of some sperm might populate the oocyte-cytoplasm, thus introducing variation into an individual. The transfer of sperm mitochondria into the cytoplasm of the oocyte is known to occur in some trematodes such as *Gonapodasmius* and *Paragonimus* [73, 74], and might, to some extent, be removed by autophagy triggered by fertilisation, as has been described for the hermaphroditic (free-living) nematode *Caenorhabditis elegans* [69, 75], which might, in turn, result in a ‘recognition failure’ and a paternal leakage of mitochondrial DNA [68]. Although challenging to address in a hermaphroditic parasite with an indirect life cycle, these aspects warrant investigation in *C*. *sinensis* to better understand its reproductive biology (fertilisation process) and the inheritance of mitochondrial DNA, which could have broader implications for other trematodes. It is possible that heteroplasmy within *C*. *sinensis* is more pronounced than currently recognised and that it may display intricate patterns within individuals (possibly allele- or tissue-specificity; [66, 67]). Future studies might utilise deep-sequencing and a double-strain validation approach [76] to elucidate levels of heteroplasmy within *C*. *sinensis*, and single-cell or single-mitochondrion sequencing [77] to elucidate intra-mitochondrial and tissue-specific patterns of mitochondrial genetic variation in this parasite.

In the present study, numerous fixed nucleotide variations were detected between some individuals of *C*. *sinensis*, which might reflect an ‘adaptive capacity’ in this species, particularly for factors linked to the establishment of infection. It is generally accepted that the adaptive capacity of a species is reflected in the levels of genetic diversity within populations, as a high level of variation provides a mechanism to respond to different selection pressures in the environment; such variation might be reflected in phenotypic variation. Phenotypic traits of particular interest in parasites, relevant to understanding parasite biology and potentially parasitic diseases, include differences in morphology, immune modulation or evasion, host preference or affiliation, sensitivity to drugs, virulence or pathogenicity, and/or adaptation to geographical regions with particular climatic conditions or host spectra. Investigating the impact that genetic variation within *C*. *sinensis* populations has on such traits relies on comprehensive genetic data and relevant biological, ecological, epidemiological or clinical information available upon sample collection. Our current data sets enabled us to investigate whether a genetic composition of adult *C*. *sinensis* populations correlated with geographical and/or known naturally infected definitive host species (cat or dog), as the worm isolates were obtained from disparate northern and southern zones in which *C*. *sinensis* is endemic [4].

We found no unequivocal mitochondrial genetic distinction between or among the geographically distinct sampling locations in ‘northern’ and ‘southern’ endemic regions, or between most other geographical sub-populations (Figs 1 and 2; S3 Table). The population from Hunan was genetically unique with respect to other provinces in China, and some haplogroups appeared to be specific to the ‘south’ (e.g., C, F and G) or ‘north’ (I) (Fig 1). However, we cannot exclude an effect of sampling bias on the findings. The apparent lack of genetic differentiation between/among most geographical regions, particularly between the ‘south’ and ‘north’, suggests that the population genetic make-up of *C*. *sinensis* is similar across the distribution range of this species, which would suggest high levels of ongoing gene-flow between and among populations, probably due to the migration of humans and/or domestic or wild canids or felids, and possibly the transport of intermediate hosts or infected food products. This is concerning in the sense that, if new, evolutionarily advantaged phenotypes emerged (e.g., genetic variants less sensitive to anthelmintics), there would be a marked potential for such variants to be spread across the distribution range of *C*. *sinensis*; it might also facilitate and enhance parasite transmission or dissemination in endemic or newly established foci of infection. It is also interesting to note that the apparent endemic hot-spots for *C*. *sinensis* in the ‘north’ and ‘south’ might differ in their epidemiology (transmission and prevalence) linked to climatic conditions (temperature, rainfall and humidity), known to have a significant impact on the success of trematode transmission, presence, prevalence and biology of intermediate host species, such as higher temperatures enhancing snail growth, activity and survival [10, 78, 79]. Whether such ecological aspects relate to differing selection pressures on *C*. *sinensis* in these regions and what impact high human migration rates might have on these possible local adaptations (cf. [80]) remain to be assessed in the future.

The lack of a clear genetic differentiation between *C*. *sinensis* from cats and dogs appears to be indicative of a plasticity in adaptation to definitive host species. Not only were the samples obtained from the two host species genetically similar, remarkably, several of them were genetically identical in their mitochondrial protein-encoding sequences (Fig 1), which suggests that the same *C*. *sinensis* variant can infect both cat and dog, and that there is likely no specific affiliation to either host species. However, we have no precise information on how transmission takes place in nature, i.e. whether genetically similar or identical worms in cats and dogs transmit through the same intermediate host species. Thus, based on current knowledge, we do not know whether genetic plasticity relating to cat and dog hosts extends to the infection of other hosts, or whether genetic variants display particular levels of host specificity. The genetic determinants of transmission and host-specificity in *C*. *sinensis* could potentially relate to a complex network of associations, as this parasite is known to infect several other ‘natural’ definitive hosts (e.g., humans, badgers, weasels and mink), in addition to cats and dogs [81], and a wide range of snail and fish intermediate host species [7, 8], whereas differences in host genotypes could also play a role. Although challenging, it would be interesting to investigate genetic variants of *C*. *sinensis* in humans to establish whether there is a genetic differentiation between isolates from people and other host species, and whether particular genotypes/haplotypes have an association with the pathogenesis, the severity of disease and complications, such as cancer, in humans. Such an endeavour would be reliant on obtaining worms during surgery, which might be feasible in endemic regions, such as southern China (e.g., Guangdong), and the Russian Far East. Alternatively, eggs from human faecal samples could be collected during epidemiological studies and subjected to genetic analysis–preferably at an individual egg level. Given the knowledge gaps regarding the genetic composition of *C*. *sinensis* in human populations, finding unique variants of *C*. *sinensis* could have significant implications for transmission and control (cf. [82]). It seems reasonable to assume that the levels of genetic diversity would correlate negatively with transmission intensity, reflecting the success of control efforts. It would also be interesting to see whether repeated praziquantel treatment of people with *C*. *sinensis* infection induces drug-resistant phenotypes of the parasite with reference to untreated populations.

Clearly, the finding of marked genetic variability within *C*. *sinensis* in the present study and the establishment of an integrated deep-sequencing-informatic-phylogenetic approach provide a solid foundation to tackle these research questions or areas. Clear proof-of-principle of this technological approach also opens the door to large-scale and comparative population genetic and molecular epidemiological analyses using whole mitochondrial and nuclear genomic data sets.

## Supporting information

S1 Table Information on the *Clonorchis sinensis* specimens (n = 183) used in this study, including their host and geographical origins as well as GenBank database accession nos. for the 12 mitochondrial protein gene sequences defined for individual specimens. (DOCX)

S2 Table Pairwise fixation index (*F*_ST_) values between haplogroups A to L inferred based on phylogenetic analyses. (DOCX)

S3 Table Pairwise fixation (*F*_ST_) indices for populations of *Clonorchis sinensis* from Russia (Primorsky Krai region), and China (Heilongjiang, Jilin, Guangdong, Guangxi and Hunan provinces). (DOCX)

S1 Fig Phylogenetic tree. This tree was constructed using concatentated sequences for the 12 mitochondrial protein genes representing 183 *Clonorchis sinensis* individuals studied here and four published mitochondrial genomes (GenBank accession nos. JF729303, FJ381664, JF729304 and KY564177) employing Bayesian (BI) and maximum likehood (ML) methods. Specimen numbers are indicated (see S1 Table); ‘s’ stands for sample. Haplotype S242 represents a *C*. *sinensis* specimen from a hamster experimentally infected with metacercariae obtained from cyprinid fish from Russia (FJ381664; [25]), haplotype S243 is from a cat from China (accession no. JF729303; [26]), haplotype S244 is from a cat from Korea (JF729304; [26]), and haplotype S245 is from a hamster experimentally infected with metacercariae obtained from cyprinid fish from South Korea (KY564177; [27]). Clusters A to L correspond to haplogroups in Fig 1; a grey outline indicates a haplogroup supported by nucleotide sequence data; a beige outline indicates a haplogroup supported by both nucleotide and amino acid sequence data. Posterior probability values (pp) are indicated by black, grey, solid or dashed lines; bootstrap support (bs) values are indicated on nodes (coloured circles). Outgroup not included in the figure.
(TIF)
