# Supplementary material for: Marked mitochondrial genetic variation in individuals and populations of the carcinogenic liver fluke Clonorchis sinensis
Source: PLoS Negl Trop Dis. 2020 Aug 19;14(8):e0008480. doi: 10.1371/journal.pntd.0008480 (PMC7437864; doi:10.1371/journal.pntd.0008480)
Supplement: S1 Table — (DOCX) [file pntd.0008480.s001.docx]

**S1 Table.** **Information on the *Clonorchis sinensis* specimens (n = 183) used in this study, including their host and geographical origins as well as GenBank database accession nos. for the 12 mitochondrial protein gene sequences defined for individual specimens.**

| **Specimen no.** | **Host species** | **Geographical origin** | **GenBank accession no.** |
| --- | --- | --- | --- |
| 1 | Cat | Guangzhou, Guangdong, China | MT292110 |
| 2 | Cat | Guangzhou, Guangdong, China | MT292111 |
| 3 | Cat | Guangzhou, Guangdong, China | MT292112 |
| 4 | Cat | Guangzhou, Guangdong, China | MT292113 |
| 5 | Cat | Guangzhou, Guangdong, China | MT292114 |
| 6 | Cat | Guangzhou, Guangdong, China | MT292115 |
| 7 | Cat | Guangzhou, Guangdong, China | MT292116 |
| 8 | Cat | Guangzhou, Guangdong, China | MT292117 |
| 9 | Cat | Guangzhou, Guangdong, China | MT292118 |
| 11 | Cat | Guangzhou, Guangdong, China | MT292119 |
| 12 | Cat | Guangzhou, Guangdong, China | MT292120 |
| 13 | Cat | Guangzhou, Guangdong, China | MT292121 |
| 15 | Cat | Guangzhou, Guangdong, China | MT292122 |
| 17 | Cat | Guangzhou, Guangdong, China | MT292123 |
| 18 | Cat | Guangzhou, Guangdong, China | MT292124 |
| 21 | Cat | Guangzhou, Guangdong, China | MT292125 |
| 22 | Cat | Guangzhou, Guangdong, China | MT292126 |
| 23 | Cat | Guangzhou, Guangdong, China | MT292127 |
| 30 | Cat | Guangzhou, Guangdong, China | MT292128 |
| 31 | Cat | Guangzhou, Guangdong, China | MT292129 |
| 32 | Cat | Guangzhou, Guangdong, China | MT292130 |
| 33 | Cat | Guangzhou, Guangdong, China | MT292131 |
| 34 | Cat | Guangzhou, Guangdong, China | MT292132 |
| 35 | Cat | Guangzhou, Guangdong, China | MT292133 |
| 36 | Cat | Guangzhou, Guangdong, China | MT292134 |
| 37 | Cat | Guangzhou, Guangdong, China | MT292135 |
| 38 | Cat | Jiangmen, Guangdong, China | MT292136 |
| 42 | Cat | Jiangmen, Guangdong, China | MT292137 |
| 43 | Cat | Jiangmen, Guangdong, China | MT292138 |
| 44 | Cat | Jiangmen, Guangdong, China | MT292139 |
| 45 | Cat | Jiangmen, Guangdong, China | MT292140 |
| 46 | Cat | Jiangmen, Guangdong, China | MT292141 |
| 47 | Cat | Jiangmen, Guangdong, China | MT292142 |
| 48 | Cat | Jiangmen, Guangdong, China | MT292143 |
| 49 | Cat | Jiangmen, Guangdong, China | MT292144 |
| 50 | Cat | Jiangmen, Guangdong, China | MT292145 |
| 51 | Cat | Shantou, Guangdong, China | MT292146 |
| 52 | Cat | Shantou, Guangdong, China | MT292147 |
| 53 | Dog | Shantou, Guangdong, China | MT292148 |
| 55 | Dog | Shantou, Guangdong, China | MT292149 |
| 56 | Dog | Xinxing, Guangdong, China | MT292150 |
| 57 | Dog | Xinxing, Guangdong, China | MT292151 |
| 58 | Dog | Xinxing, Guangdong, China | MT292152 |
| 59 | Dog | Xinxing, Guangdong, China | MT292153 |
| 60 | Dog | Xinxing, Guangdong, China | MT292154 |
| 61 | Dog | Xinxing, Guangdong, China | MT292155 |
| 62 | Dog | Xinxing, Guangdong, China | MT292156 |
| 63 | Dog | Xinxing, Guangdong, China | MT292157 |
| 64 | Dog | Xinxing, Guangdong, China | MT292158 |
| 66 | Cat | Xinxing, Guangdong, China | MT292159 |
| 67 | Cat | Xinxing, Guangdong, China | MT292160 |
| 68 | Cat | Xinxing, Guangdong, China | MT292161 |
| 69 | Cat | Xinxing, Guangdong, China | MT292162 |
| 70 | Cat | Xinxing, Guangdong, China | MT292163 |
| 72 | Cat | Foshan, Guangdong, China | MT292164 |
| 73 | Cat | Foshan, Guangdong, China | MT292165 |
| 75 | Cat | Foshan, Guangdong, China | MT292166 |
| 76 | Cat | Foshan, Guangdong, China | MT292167 |
| 77 | Cat | Foshan, Guangdong, China | MT292168 |
| 78 | Cat | Foshan, Guangdong, China | MT292169 |
| 81 | Cat | Foshan, Guangdong, China | MT292170 |
| 82 | Dog | Foshan, Guangdong, China | MT292171 |
| 83 | Dog | Foshan, Guangdong, China | MT292172 |
| 84 | Dog | Foshan, Guangdong, China | MT292173 |
| 85 | Dog | Foshan, Guangdong, China | MT292174 |
| 86 | Dog | Foshan, Guangdong, China | MT292175 |
| 87 | Dog | Foshan, Guangdong, China | MT292176 |
| 88 | Dog | Foshan, Guangdong, China | MT292177 |
| 89 | Dog | Foshan, Guangdong, China | MT292178 |
| 91 | Dog | Kaiping, Guangdong, China | MT292179 |
| 93 | Dog | Kaiping, Guangdong, China | MT292180 |
| 96 | Dog | Kaiping, Guangdong, China | MT292181 |
| 98 | Dog | Zhaoqing, Guangdong, China | MT292182 |
| 99 | Dog | Zhaoqing, Guangdong, China | MT292183 |
| 100 | Dog | Zhaoqing, Guangdong, China | MT292184 |
| 101 | Dog | Zhaoqing, Guangdong, China | MT292185 |
| 102 | Dog | Zhaoqing, Guangdong, China | MT292186 |
| 105 | Cat | Yangchun, Guangdong, China | MT292187 |
| 106 | Cat | Yangchun, Guangdong, China | MT292188 |
| 107 | Cat | Yangchun, Guangdong, China | MT292189 |
| 108 | Cat | Yangchun, Guangdong, China | MT292190 |
| 109 | Cat | Yangchun, Guangdong, China | MT292191 |
| 110 | Cat | Yangchun, Guangdong, China | MT292192 |
| 111 | Cat | Yangchun, Guangdong, China | MT292193 |
| 112 | Cat | Yangchun, Guangdong, China | MT292194 |
| 113 | Cat | Yangchun, Guangdong, China | MT292195 |
| 114 | Cat | Yangchun, Guangdong, China | MT292196 |
| 115 | Cat | Yangchun, Guangdong, China | MT292197 |
| 116 | Dog | Shenzhen, Guangdong, China | MT292198 |
| 117 | Dog | Shenzhen, Guangdong, China | MT292199 |
| 118 | Dog | Shenzhen, Guangdong, China | MT292200 |
| 119 | Dog | Shenzhen, Guangdong, China | MT292201 |
| 121 | Dog | Shenzhen, Guangdong, China | MT292202 |
| 122 | Dog | Shenzhen, Guangdong, China | MT292203 |
| 123 | Dog | Shenzhen, Guangdong, China | MT292204 |
| 124 | Dog | Shenzhen, Guangdong, China | MT292205 |
| 125 | Dog | Shenzhen, Guangdong, China | MT292206 |
| 126 | Dog | Shenzhen, Guangdong, China | MT292207 |
| 127 | Dog | Shenzhen, Guangdong, China | MT292208 |
| 128 | Dog | Meizhou, Guangdong, China | MT292209 |
| 129 | Dog | Meizhou, Guangdong, China | MT292210 |
| 130 | Dog | Meizhou, Guangdong, China | MT292211 |
| 131 | Dog | Meizhou, Guangdong, China | MT292212 |
| 134 | Dog | Meizhou, Guangdong, China | MT292213 |
| 135 | Dog | Meizhou, Guangdong, China | MT292214 |
| 136 | Dog | Meizhou, Guangdong, China | MT292215 |
| 137 | Dog | Meizhou, Guangdong, China | MT292216 |
| 138 | Dog | Meizhou, Guangdong, China | MT292217 |
| 139 | Dog | Meizhou, Guangdong, China | MT292218 |
| 140 | Dog | Shaoguan, Guangdong, China | MT292219 |
| 141 | Dog | Shaoguan, Guangdong, China | MT292220 |
| 143 | Dog | Shaoguan, Guangdong, China | MT292221 |
| 144 | Dog | Shaoguan, Guangdong, China | MT292222 |
| 145 | Dog | Shaoguan, Guangdong, China | MT292223 |
| 146 | Dog | Shaoguan, Guangdong, China | MT292224 |
| 150 | Dog | Shaoguan, Guangdong, China | MT292225 |
| 151 | Dog | Shaoguan, Guangdong, China | MT292226 |
| 152 | Dog | Shaoguan, Guangdong, China | MT292227 |
| 153 | Dog | Shaoguan, Guangdong, China | MT292228 |
| 154 | Dog | Shaoguan, Guangdong, China | MT292229 |
| 155 | Dog | Shaoguan, Guangdong, China | MT292230 |
| 156 | Dog | Shaoguan, Guangdong, China | MT292231 |
| 157 | Dog | Shaoguan, Guangdong, China | MT292232 |
| 158 | Dog | Shaoguan, Guangdong, China | MT292233 |
| 159 | Dog | Shaoguan, Guangdong, China | MT292234 |
| 160 | Dog | Shaoguan, Guangdong, China | MT292235 |
| 162 | Dog | Shaoguan, Guangdong, China | MT292236 |
| 167 | Dog | Shaoguan, Guangdong, China | MT292237 |
| 168 | Dog | Shaoguan, Guangdong, China | MT292238 |
| 169 | Dog | Shaoguan, Guangdong, China | MT292239 |
| 171 | Dog | Shaoguan, Guangdong, China | MT292240 |
| 172 | Cat | Nanning, Guangxi, China | MT292241 |
| 174 | Cat | Nanning, Guangxi, China | MT292242 |
| 176 | Cat | Nanning, Guangxi, China | MT292243 |
| 177 | Cat | Nanning, Guangxi, China | MT292244 |
| 178 | Cat | Nanning, Guangxi, China | MT292245 |
| 179 | Cat | Nanning, Guangxi, China | MT292246 |
| 180 | Cat | Nanning, Guangxi, China | MT292247 |
| 181 | Cat | Nanning, Guangxi, China | MT292248 |
| 182 | Cat | Hepu, Guangxi, China | MT292249 |
| 183 | Cat | Hepu, Guangxi, China | MT292250 |
| 184 | Cat | Hepu, Guangxi, China | MT292251 |
| 185 | Cat | Hepu, Guangxi, China | MT292252 |
| 186 | Cat | Hepu, Guangxi, China | MT292253 |
| 187 | Dog | Changsha, Hunan, China | MT292254 |
| 188 | Dog | Changsha, Hunan, China | MT292255 |
| 189 | Dog | Changsha, Hunan, China | MT292256 |
| 190 | Dog | Changsha, Hunan, China | MT292257 |
| 191 | Dog | Changsha, Hunan, China | MT292258 |
| 192 | Dog | Daqing, Heilongjiang, China | MT292259 |
| 193 | Dog | Daqing, Heilongjiang, China | MT292260 |
| 194 | Dog | Daqing, Heilongjiang, China | MT292261 |
| 195 | Dog | Daqing, Heilongjiang, China | MT292262 |
| 196 | Dog | Daqing, Heilongjiang, China | MT292263 |
| 197 | Dog | Daqing, Heilongjiang, China | MT292264 |
| 198 | Dog | Daqing, Heilongjiang, China | MT292265 |
| 199 | Dog | Daqing, Heilongjiang, China | MT292266 |
| 200 | Dog | Daqing, Heilongjiang, China | MT292267 |
| 211 | Dog | Fuyu, Jilin, China | MT292268 |
| 212 | Dog | Fuyu, Jilin, China | MT292269 |
| 214 | Dog | Fuyu, Jilin, China | MT292270 |
| 215 | Dog | Fuyu, Jilin, China | MT292271 |
| 216 | Dog | Fuyu, Jilin, China | MT292272 |
| 217 | Dog | Fuyu, Jilin, China | MT292273 |
| 218 | Dog | Fuyu, Jilin, China | MT292274 |
| 219 | Dog | Fuyu, Jilin, China | MT292275 |
| 220 | Dog | Fuyu, Jilin, China | MT292276 |
| 221 | Dog | Fuyu, Jilin, China | MT292277 |
| 222 | Dog | Fuyu, Jilin, China | MT292278 |
| 223 | Dog | Fuyu, Jilin, China | MT292279 |
| 224 | Dog | Fuyu, Jilin, China | MT292280 |
| 226 | Cyprinid fish^a^ | Ilystaya river, Primorsky Krai, Russia | MT292281 |
| 227 | Cyprinid fish^a^ | Ilystaya river, Primorsky Krai, Russia | MT292282 |
| 228 | Cyprinid fish^a^ | Ilystaya river, Primorsky Krai, Russia | MT292283 |
| 229 | Cyprinid fish^a^ | Komarovka river, Primorsky Krai, Russia | MT292284 |
| 230 | Cyprinid fish^a^ | Komarovka river, Primorsky Krai, Russia | MT292285 |
| 232 | Cyprinid fish^a^ | Kronshtadtka river, Primorsky Krai, Russia | MT292286 |
| 233 | Cyprinid fish^a^ | Kronshtadtka river, Primorsky Krai, Russia | MT292287 |
| 234 | Cyprinid fish^a^ | Kronshtadtka river, Primorsky Krai, Russia | MT292288 |
| 238 | Cyprinid fish^a^ | Soldatskoe lake, Primorsky Krai, Russia | MT292289 |
| 239 | Cyprinid fish^a^ | Soldatskoe lake, Primorsky Krai, Russia | MT292290 |
| 240 | Cyprinid fish^a^ | Soldatskoe lake, Primorsky Krai, Russia | MT292291 |
| 241 | Cyprinid fish^a^ | Soldatskoe lake, Primorsky Krai, Russia | MT292292 |

^a^ Specimens from rats experimentally infected with metacercariae from naturally infected cyprinid fish.
