# Supplementary material for: Marked mitochondrial genetic variation in individuals and populations of the carcinogenic liver fluke Clonorchis sinensis
Source: PLoS Negl Trop Dis. 2020 Aug 19;14(8):e0008480. doi: 10.1371/journal.pntd.0008480 (PMC7437864; doi:10.1371/journal.pntd.0008480)
Supplement: S2 Table — (DOCX) [file pntd.0008480.s002.docx]

**S2 Table.** **Pairwise fixation index (*F*_ST_) values between haplogroups A to L inferred based on phylogenetic analyses.**

|  | A | B | C | D | E | F | G | H | I | J | K | L |
| --- | --- | --- | --- | --- | --- | --- | --- | --- | --- | --- | --- | --- |
| A | - |  |  |  |  |  |  |  |  |  |  |  |
| B | 0.43692^a^ | - |  |  |  |  |  |  |  |  |  |  |
| C | 0.61929^a^ | 0.61919^a^ | - |  |  |  |  |  |  |  |  |  |
| D | 0.51898^a^ | 0.49577^a^ | 0.57875^a^ | - |  |  |  |  |  |  |  |  |
| E | 0.60416^a^ | 0.58921^a^ | 0.66265^a^ | 0.59081^a^ | - |  |  |  |  |  |  |  |
| F | 0.56635^a^ | 0.56262^a^ | 0.65777^a^ | 0.55673^a^ | 0.51809^a^ | - |  |  |  |  |  |  |
| G | 0.57469^a^ | 0.55534^a^ | 0.63640^a^ | 0.54457^a^ | 0.59876^a^ | 0.57259^a^ | - |  |  |  |  |  |
| H | 0.61380^a^ | 0.60171^a^ | 0.65441^b^ | 0.59044^a^ | 0.64244^a^ | 0.60392^a^ | 0.59932^a^ | - |  |  |  |  |
| I | 0.62842^a^ | 0.62998^a^ | 0.75024^b^ | 0.60577^a^ | 0.65485^a^ | 0.65966^a^ | 0.63931^a^ | 0.66292^a^ | - |  |  |  |
| J | 0.72842^a^ | 0.72827^a^ | 0.78072^b^ | 0.68764^a^ | 0.74254^a^ | 0.74325^a^ | 0.71514^b^ | 0.73024^b^ | 0.80709^b^ | - |  |  |
| K | 0.69460^a^ | 0.68862^a^ | 0.73128^b^ | 0.67049^a^ | 0.71352^a^ | 0.70877^a^ | 0.68228^a^ | 0.69707^b^ | 0.76366^b^ | 0.75701^c^ |  |  |
| L | 0.56636^a^ | 0.53625^a^ | 0.57500^b^ | 0.53685^a^ | 0.60075^a^ | 0.56736^a^ | 0.50035^a^ | 0.56195^a^ | 0.59802^b^ | 0.65235^b^ | 0.61757^b^ | - |

^a^ *p* value < 0.001. ^b^ *p* value < 0.01. ^c^ *p* value < 0.05.
