# Supplementary material for: Marked mitochondrial genetic variation in individuals and populations of the carcinogenic liver fluke Clonorchis sinensis
Source: PLoS Negl Trop Dis. 2020 Aug 19;14(8):e0008480. doi: 10.1371/journal.pntd.0008480 (PMC7437864; doi:10.1371/journal.pntd.0008480)
Supplement: S3 Table — (DOCX) [file pntd.0008480.s003.docx]

**S3 Table. Pairwise fixation (*F*_ST_) indices for populations of *Clonorchis sinensis* from Russia (Primorsky Krai region), and China (Heilongjiang, Jilin, Guangdong, Guangxi and Hunan provinces).**

|  | Primorsky Krai | Heilongjiang | Jilin | Guangdong | Guangxi | Hunan |
| --- | --- | --- | --- | --- | --- | --- |
| Primorsky Krai | - |  |  |  |  |  |
| Heilongjiang | 0.04596^d^ | - |  |  |  |  |
| Jilin | 0.07941^c^ | 0.08150^c^ | - |  |  |  |
| Guangdong | 0.03943^b^ | 0.06600^a^ | 0.03916^a^ |  |  |  |
| Guangxi | 0.09999^a^ | 0.07094^b^ | 0.09894^a^ | 0.02364^c^ | - |  |
| Hunan | 0.46279^a^ | 0.44254^a^ | 0.56811^a^ | 0.42205^a^ | 0.45950^a^ | - |

^a^ *p* value (p < 0.001). ^b^ *p* value (p < 0.01). ^c^ *p* value (p < 0.05). ^d^ *p* value (p > 0.05).
