# Supplementary figures and images for: Marked mitochondrial genetic variation in individuals and populations of the carcinogenic liver fluke Clonorchis sinensis
Source: PLoS Negl Trop Dis. 2020 Aug 19;14(8):e0008480. doi: 10.1371/journal.pntd.0008480 (PMC7437864; doi:10.1371/journal.pntd.0008480)

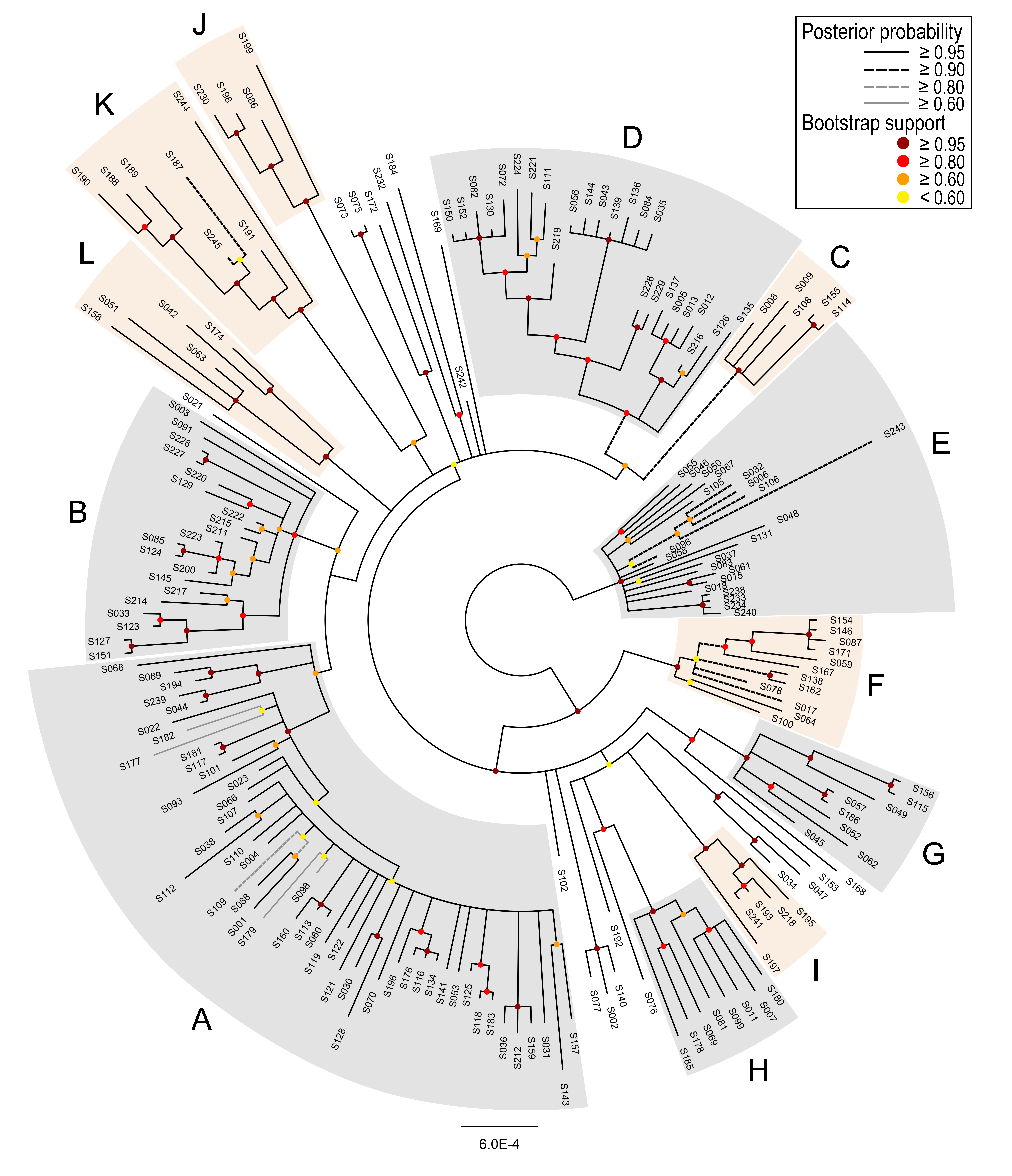

Supplement: S1 Fig — This tree was constructed using concatentated sequences for the 12 mitochondrial protein genes representing 183 Clonorchis sinensis individuals studied here and four published mitochondrial genomes (GenBank accession nos. JF729303, FJ381664, JF729304 and KY564177) employing Bayesian (BI) and maximum likehood (ML) methods. Specimen numbers are indicated (see S1 Table); ‘s’ stands for sample. Haplotype S242 represents a C. sinensis specimen from a hamster experimentally infected with metacercariae obtained from cyprinid fish from Russia (FJ381664; [25]), haplotype S243 is from a cat from China (accession no. JF729303; [26]), haplotype S244 is from a cat from Korea (JF729304; [26]), and haplotype S245 is from a hamster experimentally infected with metacercariae obtained from cyprinid fish from South Korea (KY564177; [27]). Clusters A to L correspond to haplogroups in Fig 1; a grey outline indicates a haplogroup supported by nucleotide sequence data; a beige outline indicates a haplogroup supported by both nucleotide and amino acid sequence data. Posterior probability values (pp) are indicated by black, grey, solid or dashed lines; bootstrap support (bs) values are indicated on nodes (coloured circles). Outgroup not included in the figure. (TIF) [file pntd.0008480.s004.tif]
